# Type of Tea Consumption and Mild Cognition Impairment in Older Adults

**DOI:** 10.1093/geroni/igaa057.949

**Published:** 2020-12-16

**Authors:** Yao Yao, Huashuai Chen, Danan Gu, Yi Zeng

**Affiliations:** 1 Peking University, Durham, North Carolina, United States; 2 Duke University, Durham, North Carolina, United States; 3 United Nations, New York, New York, United States

## Abstract

Existing studies have testified the neuroprotective qualities of tea. As there are several types of tea, question on which type of tea may exert substantial influence on cognitive health is intriguing and remains unknow. We aim to estimate the association between type of tea consumption and mild cognition impairment (MCI) using a nationally representative dataset of older population in China. Type of tea consumption was classified as three groups: Green, fermented (White, Oolong, Black, and Pu’eh), and flower tea. The Mini-Mental State Examination (MMSE) was adopted to assess cognitive function. We conducted multivariate logistic regressions to evaluate the association between type of tea drinking and cognition outcomes (MMSE score and MCI). Potential confounders including sociodemographic factors, health conditions, dietary patterns, lifestyles, activities of daily living, mental health, and living environments. A total of 10,923 participants (mean age: 85.4 yr; female: 53.5%) included in the study. The type of current tea consumption among the participants were: 2143 for green tea, 1302 for fermented tea, and 844 for flower tea. Compared to those who had no habit of tea consumption, the odds ratio of MCI in green tea drinkers was 0.80 (0.68-0.95), in fermented tea drinkers was 1.07 (0.89-1.30), and in flower tea drinkers were 0.85 (0.67-1.09). Our study showed green tea and flower tea consumption associated with lower odds of MCI, while the association was not found among fermented tea drinkers. Future experimental and longitudinal studies are warranted to illustrate the association between varied type of tea and cognitive health.

